# When violence meets leadership: Leadership climate as a buffer in the link between violence, harmful alcohol use, and burnout among healthcare workers in Sweden

**DOI:** 10.1016/j.ijnsa.2026.100600

**Published:** 2026-06-15

**Authors:** Josefina Peláez Zuberbuhler, Emelie Thern, Bodil J. Landstad, Malin Sjöström, Emma Brulin

**Affiliations:** a Department of Leadership and Organization, Kristiania University of Applied Sciences, Oslo, Norway; b Unit of Occupational Medicine, Institute of Environmental Medicine, Karolinska Institutet, Solna, Sweden; c Department of Health Sciences, Division of Public Health Science, Mid Sweden University, Östersund, Sweden; d Unit of Research, Östersund Hospital, Östersund, Sweden; e Department of Public Health and Clinical Medicine, Umeå University, Umeå, Sweden; f Center for Occupational and Environmental Medicine, Region Stockholm, Stockholm, Sweden

**Keywords:** Leadership climate, Workplace violence, Harmful alcohol use, Burnout, Healthcare workers

## Abstract

**Background:**

Healthcare workers are frequently exposed to workplace violence (including threats and physical aggression), which may increase the risk of burnout complaints through maladaptive coping behaviours such as harmful alcohol use. Leadership climate may buffer these adverse effects by fostering psychological safety and supportive supervisor–employee interactions. This study examined whether leadership climate moderates the association between violence and harmful alcohol use, whether the indirect association between violence and subsequent burnout via harmful alcohol use depends on leadership climate, and whether these pathways differ between physicians and nurses.

**Methods:**

Two-wave panel data were drawn from the Longitudinal Occupational Health Survey in Healthcare Sweden (LOHHCS). The sample included 2446 healthcare workers. Violence, harmful alcohol use, and leadership climate were assessed at Time 1, while burnout was measured at Time 2. Moderated mediation analyses were conducted using the PROCESS macro.

**Results:**

Violence was positively associated with harmful alcohol use, with this association weakened under stronger leadership climate. The conditional indirect prospective association between violence and later burnout through harmful alcohol use was evident primarily under weaker leadership conditions (b = 0.007, 95% CI [0.001, 0.016]) and nonsignificant under moderate or strong leadership. Formal tests did not indicate statistically significant differences between professional groups.

**Conclusions:**

A supportive leadership climate may play a modest protective role in the associations between violence and harmful alcohol use and subsequent burnout. Despite small effect sizes and limited causal inference, consistency with theory suggests that leadership climate may play a meaningful protective role in violence-exposed healthcare environments.

**Registration:**

Swedish Occupational and Education Registries

**What is already known**

• Workplace violence is linked to higher burnout in healthcare workers.
• Work stressors can increase harmful alcohol use as a coping response.
• Supportive leadership is associated with better well-being, but its role in the violence→alcohol→burnout pathway is unclear.

Alt-text: Unlabelled box dummy alt text

**What this paper adds**

• Violence was positively associated with harmful alcohol use.
• Leadership climate weakened the violence–harmful alcohol use association.
• The indirect violence→burnout association was evident primarily under weaker leadership climate.

Alt-text: Unlabelled box dummy alt text

## 1 Background

Healthcare workers in Sweden face significant occupational stressors, including exposure to workplace violence (ranging from verbal threats to physical aggression) from patients, co-workers, or the public, which can negatively affect their mental health, job satisfaction, and retention (Arnetz and Arnetz, 2001; Pariona‐Cabrera, Cavanagh, and Bartram, 2020). Recent reviews and international reports show that workplace violence against healthcare workers continues to rise worldwide, particularly in healthcare settings, posing serious threats to psychological safety and workforce sustainability (O’Brien, Van Zundert, and Barach 2024; Yusoff et al., 2023). Such stressors are not only psychologically taxing but may also increase vulnerability to maladaptive coping behaviours, such as harmful alcohol use, i.e., drinking consumption that has caused damage to health (Ewing, 1984), which in turn can contribute to emotional exhaustion and burnout (O’Brien et al., 2024; Teo, Bentley, and Nguyen 2020).

In Sweden and other European countries, workplace threats or violence have been recognised as an increasing occupational health challenge. Healthcare workers in Sweden are now more exposed to violence than employees in other high-risk professions, including law enforcement (Mento et al., 2020). A review of 32 studies found that the prevalence of threats or violence at work among healthcare workers ranged from 30% to 43% (Rossi et al., 2023), and more recent meta-analyses report that up to half of healthcare workers worldwide experience some form of workplace violence each year (O’Brien et al., 2024; Yusoff et al., 2023), potentially leading to higher levels of burnout and emotional exhaustion among healthcare workers (Lanctôt and Guay, 2014;).

Building on this evidence, previous longitudinal research using the same LOHHCS cohort as in this study found that exposure to threats and violence was prospectively associated with a higher risk of problem drinking at follow-up among physicians in Sweden, even after adjusting for other forms of workplace mistreatment (Peláez Zuberbuhler et al., 2026). These findings highlight the potential for maladaptive coping behaviours, such as harmful alcohol use, to emerge as behavioural responses to workplace violence. This hypothesis is supported by broader occupational health research showing that exposure to interpersonal conflict, harassment, and related work stressors is associated with increased alcohol use as a coping response (Frone, 2016; Richman et al., 2001).

The Job Demands–Resources (JD–R) theory (Bakker et al., 2023) provides a useful framework for understanding why workplace violence may be associated with harmful alcohol use and later burnout complaints. Within this perspective, workplace violence can be understood as a psychosocial job demand that threatens employees’ sense of safety, increases emotional strain, and depletes coping resources over time (Bakker et al., 2023; Dollard and Bakker, 2010). When such demands are recurrent and insufficiently buffered, workers may rely on maladaptive coping strategies to manage distress. One such behavioural response may be harmful alcohol use, which has been linked in prior research to work stress, interpersonal mistreatment, and self-medication processes (Frone, 2016; Richman et al., 2001). Harmful alcohol use may, in turn, undermine recovery, impair emotion regulation, and contribute to later burnout complaints (Frone, 2016). In this way, alcohol use may represent one potential behavioural pathway through which exposure to violence becomes linked to longer-term strain. In this study, we will therefore test the path from workplace violence, harmful alcohol use and later burnout complaints.

At the same time, the JD-R model posits that job resources can buffer or mitigate the negative consequences of job demands by enhancing workers’ coping, recovery, and perceived control (Bakker et al., 2023; Dollard and Bakker, 2010; McLinton et al., 2019). One such resource is leadership climate—broadly defined as employees’ shared perceptions of leadership behaviours related to communication, fairness, and support for psychological health (Klinefelter et al., 2021; Spence Laschinger et al., 2009). Leadership climate has been consistently associated with lower stress levels, higher well-being, and greater organisational commitment (Klinefelter et al., 2021; Spence Laschinger et al., 2009). In the Swedish healthcare context, leadership climate has been identified as a particularly important organisational resource and protective factor. For instance, physicians reporting higher social support and a favourable leadership climate showed lower turnover intentions and higher job satisfaction (Fernemark et al., 2024). Similarly, supportive and empowering leadership has been linked to reduced absenteeism, improved psychological safety, and better team functioning among nurses and other healthcare staff (McLinton et al. 2019; Stengård et al. 2021). Importantly, previous research indicates that a positive leadership style can buffer the negative effects of workplace incivility on employee well-being and work-related outcomes, even when exposure to violence cannot be fully prevented (Laschinger and Read, 2016). Against this backdrop, in contexts marked by violence or threats, a more favourable leadership climate may reduce uncertainty, facilitate support-seeking, and strengthen employees’ sense that stressful events can be managed constructively, thereby promoting more adaptive coping responses (McLinton et al., 2019; Stengård et al., 2021). In contrast, weaker leadership conditions may increase vulnerability to maladaptive coping strategies, including harmful alcohol use, as suggested by research on stress and self-medication processes (Frone, 2016; Richman et al., 2001). Thus, leadership climate may operate not only as a direct organisational resource, but also as a contextual condition that weakens the association between workplace violence and harmful alcohol use, and consequently the prospective indirect association with later burnout complaints.

Furthermore, previous research suggests that physicians and nurses may differ in both their exposure to threats or violence and in their perceptions of the role of leadership climate as a protective factor (Dai et al., 2025; Friese et al., 2024) as well as in burnout levels (Brulin et al., 2023). Physicians often experience greater professional autonomy, whereas nurses are more exposed to patient aggression and more dependent on supportive leadership within team-based work structures (Arnetz and Arnetz, 2001; Fernemark et al., 2024; O’Brien et al., 2024). These occupational differences underscore the need to examine whether the strengths of the associations differ across professional groups.

Building on the JD–R framework and prior research on coping responses to occupational stress, this study examined whether: (1) violence is associated with subsequent harmful alcohol use and burnout, respectively, (2) leadership climate moderates the association between violence and harmful alcohol use, such that the association between violence and harmful alcohol use will be weaker under stronger leadership climate, (3) the indirect association between violence and burnout via harmful alcohol use varies as a function of leadership climate (i.e., a moderated mediation), and (4) these pathways differ across professional groups, with the moderated mediation expected to be stronger among nurses compared with physicians. By integrating these components, the study aims to clarify how organisational resources, such as leadership climate, may shape the prospective associations between workplace violence, harmful alcohol use as a behavioural coping response, and subsequent psychological strain.

## 2 Methods

### 2.1 Sample and data collection

Data were drawn from the Longitudinal Occupational Health Survey in Healthcare Sweden (LOHHCS), conducted in 2022 (T1) and 2023 (T2). The LOHHCS project collects data on psychosocial working conditions and health among healthcare workers through comprehensive questionnaires administered by Statistics Sweden. Participants were randomly selected from the Swedish Occupational and Education Registries to ensure a diverse and representative sample of healthcare workers across the country. The LOHHCS functions as an open cohort, meaning that at each wave of data collection, newly educated healthcare workers are randomly recruited, while individuals who have left the healthcare labour market (migrated, died, or retired) are excluded. This design ensures both representativeness and renewal of the sample over time.

In 2022, a stratified random sample of healthcare workers (including 7908 physicians and 7790 nurses) was invited to participate in the LOHHCS survey. The overall response rate at baseline was approximately 36%, yielding 2652 completed questionnaires. In fall 2023, 7780 physicians and 7634 nurses received the follow-up survey, with response rates of 36.4% and 34.0%, respectively. To focus the analyses on prospective associations with subsequent burnout complaints rather than persistence of already severe burnout, participants classified as at high risk of burnout at T1 (based on the BAT-12 cut-off ≥ 2.96) were excluded from the main analyses. This restriction was intended to reduce the likelihood that observed associations primarily reflected continuation of pre-existing severe burnout. For this study, we excluded those older than 69 years. As a result, the analytical sample consisted of 2446 healthcare workers (40% physicians, 60% nurses) who participated at both time points, and had low or moderate burnout levels at baseline.

All potential participants were informed that participation was voluntary and anonymous, and no financial compensation was provided. Data were pseudonymized by Statistics Sweden prior to delivery to the research team and stored securely in compliance with data protection regulations.

The study was approved by the Swedish Ethical Review Authority (Dnr 2020–06,613; 2021–05,574-02; 2022–00,310-02).

### 2.2 Measures

#### 2.2.1 Leadership climate

Leadership climate was assessed using a 10-item validated instrument developed by Setterlind and Larsson (1995) and later refined by Nyberg et al. (2009). Example items include *“I am clear about what my boss expects of me”* and *“My boss encourages my participation in the setup of my work.”* Response options ranged from 1 (*yes, often),* to 4 (*no, never)*, with an additional *not relevant* category. Cronbach’s α = 0.88 indicated excellent internal consistency. For interpretability, items were reverse-scored, so higher values reflected a more favourable leadership climate. Higher scores, therefore, represent a more supportive and health-promoting leadership environment.

#### 2.2.2 Workplace violence

Exposure to workplace violence was measured with a single item: *“In the past 12 months, have you been exposed to violence or threats of violence at work?”* The questions draw on the Swedish Work Environment survey (The Work Environment survey, 2025). Responses were rated on a four-point Likert scale (1 = never, 4 = one or more times per week) and dichotomised into *yes* (any exposure) and *no* (never) for analysis.

#### 2.2.3 Harmful alcohol use

Harmful alcohol use was measured using a modified version of the CAGE questionnaire (Dhalla and Kopec, 2007; Ewing, 1984). Participants first indicated frequency of alcohol consumption on a five-point scale, ranging from ‘never’ (1) to ‘every day’ (5). Those reporting any drinking beyond *never* completed the four-item CAGE (e.g., *“Have you ever felt you should cut down on your drinking?”*). Each item has a 0 (no) and 1 (yes) score. Following standard scoring, higher total scores reflect more harmful patterns of alcohol use.

#### 2.2.4 Burnout complaints

Burnout complaints were measured using the 12-item Burnout Assessment Tool (BAT-12) (Schaufeli et al., 2020). Items were rated on a five-point Likert scale (1 = never, 5 = always) and included statements such as *“When I am at work, I feel mentally exhausted.”* Cronbach’s α = 0.90, indicating excellent reliability. A grand-mean score was computed, with a higher score indicating more severe burnout complaints (Schaufeli et al., 2023).

#### 2.2.5 Covariates

Potential confounders tested at T1 included profession (physician or nurse), sex (male or female), years of work experience (< than 15 years or ≥ 15 years), and workplace setting (primary care, municipality, hospital, and other). These covariates were selected based on prior research identifying their associations with occupational stress, coping behaviours, and burnout in healthcare (Aronsson et al., 2017; Theorell et al., 2015).

#### 2.2.6 Data analysis

Descriptive statistics summarised participant characteristics and study variables. Pearson correlations were calculated to examine preliminary associations among violence, leadership climate, harmful alcohol use, burnout, and covariates. Variance inflation factors (VIFs) were examined to assess multicollinearity.

**Fig. 1:**
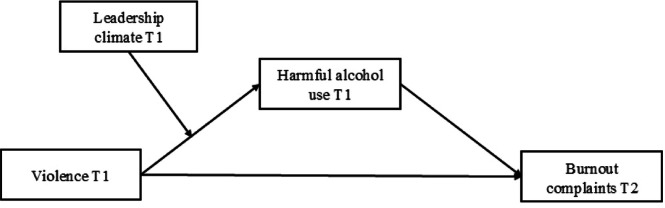
Conceptual model of the moderated mediation.

To test the hypothesised model, a moderated mediation analysis was conducted using the PROCESS macro v3.3 for SPSS (Model 7; Hayes, 2018). In this model, violence (X) was specified as a predictor of harmful alcohol use (M) and burnout (Y). Next, leadership climate (W) was modelled as a moderator of the X → M path, thereby testing whether the association between violence and harmful alcohol use varied as a function of leadership climate. This setup also tests whether the indirect association between violence and later burnout via harmful alcohol use was conditional on leadership climate (i.e., a moderated mediation). Because workplace violence, leadership climate, and harmful alcohol use were all assessed at T1, the estimated indirect associations should be interpreted as conditional indirect prospective associations rather than causal effects. The conceptual model and hypothesised pathways are depicted in Fig. 1.

Leadership climate, as a continuous moderator, was mean-centred prior to computing the interaction term. Covariates (sex, profession, years of experience, workplace) were included in both the mediator and outcome models. Bootstrap resampling (5000 samples) with bias-corrected 95% confidence intervals was used to test indirect and conditional associations. Effects were deemed significant when the confidence interval did not include zero. All tests were two-tailed with α = 0.05.

To examine whether the proposed pathways differed across professional groups, the full moderated mediation model was then estimated separately for physicians and for nurses (including assistant nurses).

## 3 Results

### 3.1 Preliminary analyses

A total of 2446 healthcare workers were included in the analytic sample after excluding participants with high burnout complaint risk at Time 1. The sample comprised physicians (40%) and nurses (60%). The majority were female (78%), and 53% had >15 years of experience. Approximately 39% reported exposure to violence in the past 12 months, and 4.3% met CAGE criteria for harmful alcohol use. Mean burnout scores at Time 2 (BAT-12) indicated moderate levels of burnout (*M* = 1.76, *SD* = 0.66). Descriptive statistics and intercorrelations appear in Tables 1 **and** 2.

**Table 1:** Sample characteristics.

	Category / Statistic	n (%) / M (SD)
Profession	Physician	1515 (40%)
	Nurse	2285 (60%)
Sex	Female	2971 (78.2%)
	Male	829 (21.8%)
Workplace	Primary care	771 (20.3%)
	Municipality	725 (19.1%)
	Hospital	1789 (47.2%)
	Other	508 (13.4%)
Years of experience	<5	511 (13.5%)
	5–10	620 (16.3)
	10–15	654 (17.2)
	>15	2009 (53%)
Exposure to violence (T1)	Yes	1208 (31.9%)
	No	2574 (68.1%)
Harmful alcohol use (T1)	Mean score	0.21 (0.53)
Leadership climate (T1)	Mean score	3.03 (0.58)
Burnout complaints (T2)	Mean score	1.76 (0.66)

**Note:** These data represent the analytic sample after exclusion of participants with high burnout at Time 1.

**Table 2:** Intercorrelations among study variables.

	1	2	3	4
1. Violence (T1)	—	−0.77*	.009	.136***
2. Leadership climate (T1)		—	−0.042*	−0.214***
3. Harmful alcohol use (T1)			—	.076***
4. Burnout complaints (T2)				—

**Note.** T1 = Time 1 (2022); T2 = Time 2 (2023); < 0.05, p < .01, p < .001 (two-tailed).

Violence was positively correlated with harmful alcohol use and burnout, respectively, whereas leadership climate was negatively correlated with both harmful alcohol use and burnout. Women and those with fewer years of experience were also significantly more likely to experience higher burnout than their counterparts. No evidence of multicollinearity was observed (all VIFs < 2.0).

### 3.2 Associations between violence, harmful alcohol use, and burnout (aim 1)

As shown in Table 3, the regression model for harmful alcohol use was statistically significant, F(7, 2499) = 6.14, *p* < .001, explaining a small proportion of the variance (R² = 0.017). Standardised coefficients (β) are reported alongside unstandardised estimates to aid interpretation of effect sizes. Higher exposure to violence was associated with a higher likelihood of more harmful alcohol use (*b* = 0.36, SE = 0.09, t = 3.97, *p* = <0.001; β = 0.41).

**Table 3:** Multiple regression predicting harmful alcohol use (T1).

	B	SE	β	t	p
Constant	0.265	0.090	—	2.95	.003
Violence (T1)	0.355	0.089	0.412	3.97	< 0.001
Leadership climate (T1)	0.008	0.022	0.009	0.38	.706
Violence × Leadership	–0.111	0.029	−0.395	–3.81	< 0.001
Profession	−0.010	0.016	−0.014	−0.63	.527
Sex (1 = woman)	–0.094	0.027	−0.076	–3.56	< 0.001
Workplace (1 = hospital)	0.027	0.011	0.050	2.49	.013
Years of experience	0.006	0.010	0.013	0.62	.538

Model summary: F(7, 2499) = 6.14, p < .001, R² = 0.017.

**Note.** b = unstandardized regression coefficient; SE = standard error; LLCI and ULCI = 95% confidence interval limits.

In the model predicting burnout complaints at Time 2 (Table 4), violence showed a statistically significant positive association with burnout complaints one year later (*b* = 0.11, SE = 0.02, *p* < .001; β = 0.12). Harmful alcohol use was also positively associated with subsequent burnout (*b* = 0.09, SE = 0.02, *p* < 0.001; β = 0.08).

**Table 4:** Multiple regression predicting burnout complaints at Time 2.

	B	SE	β	t	p
Constant	1.822	0.056	—	32.26	< 0.001
Violence (T1)	0.113	0.017	0.123	6.65	< 0.001
Harmful alcohol use (T1)	0.087	0.019	0.082	4.61	< 0.001
Profession	−0.053	0.015	−0.069	−3.44	< 0.001
Sex (1 = woman)	0.141	0.025	0.106	5.57	< 0.001
Workplace (1 = hospital)	−0.020	0.010	−0.035	−2.00	.046
Years of experience	−0.073	0.009	−0.145	−7.94	< 0.001

Model summary: F(6, 3036) = 29.92, p < .001, R² = 0.056.

**Note.** b = unstandardized regression coefficient; SE = standard error; LLCI and ULCI = 95% confidence interval limits.

### 3.3 Moderation of violence → harmful alcohol use by leadership climate (aim 2)

Next, we examined the interaction between violence and leadership climate. Leadership climate showed no main association with harmful alcohol use (*p* = .82). However, as expected, the interaction term between violence and leadership climate was statistically significant (*b* = –.12, *SE* = 0.04, *t* = –3.00, *p* = .003; β = −0.40; Table 3). The interaction term accounted for a small proportion of additional variance (ΔR² ≈ 0.003), indicating a modest effect size. Simple-slope analyses indicated that under weak leadership climate (–1 SD = 2.4), violence was statistically significantly positively associated with harmful alcohol use (*b* = 0.09, *p* = .011); whereas this association was nonsignificant under moderate (M = 3.1; (*b* = 0.00, *p* = .93) or strong (+1 SD = 3.6; (*b* = –.06, *p* = .09)) leadership climate.

### 3.4 Conditional indirect associations (moderated mediation; aim 3)

Conditional indirect associations between violence and burnout via harmful alcohol use across levels of leadership climate are summarised in Table 5. The indirect association was statistically significant only under weak leadership (Boot 95% CI [.0009, 0.0163]) and was nonsignificant at moderate or strong levels of leadership climate. The index of moderated mediation was statistically significant (*b* = –.0100, Boot95% CI [–.0203, –.0027]).

**Table 5:** Conditional indirect associations of violence (T1) on burnout complaints (T2) through harmful alcohol use (T1) at different levels of leadership climate (T1).

Leadership climate level	Effect	BootSE	BootLLCI	BootULCI
Weak (–1 SD = 2.4)	0.0072	0.0038	0.0009	0.0163
Moderate (M = 3.1)	0.0002	0.0020	–0.0036	0.0045
Strong (+1 SD = 3.6)	–0.0048	0.0028	–0.0107	0.0002

**Note.** Based on 5000 bootstrap samples. BootLLCI and BootULCI = 95% bias-corrected bootstrap.

confidence interval limits. Indirect effect is significant when the CI does not include zero.

**Fig. 2:**
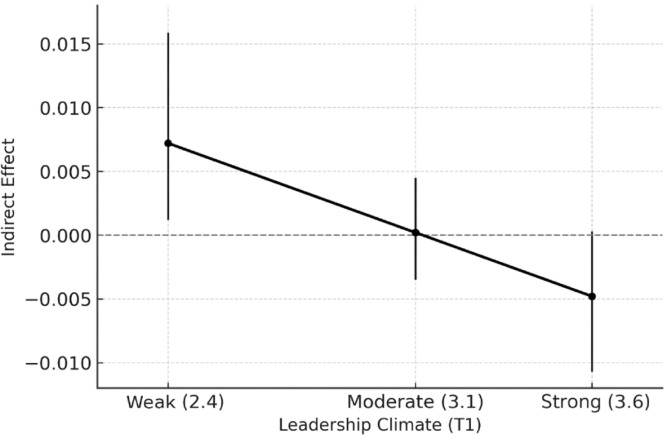
Conditional indirect association of violence with burnout through harmful use of alcohol across levels of leadership climate (–1 SD, M, +1 SD), with errors representing 95% confidence intervals.

As illustrated in Fig. 2, the indirect association was stronger under weak leadership and diminished as leadership climate increased. The figure depicts the conditional indirect associations at low (–1 SD = 2.4), moderate (mean; M = 3.1), and high (+1 SD = 3.6) levels of leadership climate, with error bars representing 95% confidence intervals.

Additional sensitivity analyses were carried out using the original four-category measure of workplace violence, which yielded a similar overall pattern. The interaction with leadership climate remained significant, but the conditional indirect effects were smaller and not consistently significant, indicating some attenuation of the moderated mediation pattern. These results should be reviewed in light of the skewed nature of the four-category violence measure, which lacks meaningful equal intervals and may dilute indirect effects in complex moderated mediation models.

### 3.5 Profession-stratified analyses (aim 4)

To explore whether the moderated mediation pathways operated similarly across professional groups, the moderated mediation model was estimated separately within each professional group. However, formal interaction tests did not indicate statistically significant differences between physicians and nurses (see Table 6).

**Table 6:** Stratified moderated mediation results by profession.

	Nurses (*n* = 1470)			Physicians (*n* = 980)		
	B	SE	p	b	SE	p
Violence → Harmful alcohol use	0.39	0.15	.013	0.30	0.20	.140
Leadership Climate → Harmful alcohol use	0.02	0.03	.540	–0.01	0.03	.820
Violence × Leadership Climate	–0.13	0.05	.011	–0.09	0.07	.160
Harmful alcohol use → Burnout complaints (T2)	0.09	0.03	.004	0.07	0.03	.020
Direct path: Violence → Burnout complaints (T2)	0.17	0.03	< 0.001	0.13	0.04	.002
Conditional Indirect Path (Weak Leadership)	0.0089	—	95% CI [–0.0011, 0.0225]	0.0027	—	95% CI [–0.0030, 0.0110]
Index of Moderated Mediation	–0.0144	0.007	95% CI [–0.0297, –0.0029]	–0.0039	0.004	95% CI [–0.0151, 0.0021]

**Note.** Conditional indirect effects are based on bias-corrected bootstrap confidence intervals (5000 samples); Dashes (—) indicate that PROCESS does not provide standard errors for bootstrap indirect effects.

Among nurses, violence significantly predicted harmful alcohol use (*b* = 0.39, SE = 0.15, *p* = .013), and this association was moderated by leadership climate (*b* = –.13, SE = 0.05, *p* = .011). The conditional indirect association of violence with burnout through harmful alcohol use was observed under weak leadership but did not reach statistical significance (effect = 0.0089, 95% CI [–.0011, 0.0225]). However, the index of moderated mediation was statistically significant (*b* = –.0144, 95% CI [–.0297, –.0029]), indicating that the indirect association varied as a function of leadership climate. In contrast, among physicians, neither the association between violence and harmful alcohol use (*b* = 0.30, *p* = .14) nor the interaction with leadership climate (*b* = –.09, *p* = .16) reached significance. Harmful alcohol use did not account for the association between violence and burnout (*b* = –.0039, 95% CI [–.0151, 0.0021]), although the direct association between violence and burnout remained statistically significant (*b* = 0.13, *p* = .002). Next, we conducted a formal three-way interaction between profession, workplace violence, and leadership climate, adjusting for all confounders, which was not statistically significant (*b* = −.02, *p* = .578), indicating that the moderation pattern did not differ significantly across professional groups.

## 4 Discussion

This two-wave panel study examined the association between workplace violence, harmful alcohol use and later burnout complaints, and whether this pathway is moderated by leadership climate among healthcare workers in Sweden. Overall, our findings were broadly consistent with the proposed model, and suggest that leadership climate may modestly attenuate the adverse behavioural and emotional consequences associated with exposure to workplace violence. However, the observed effects were modest and should be interpreted with caution.

Consistent with prior research, workplace violence was associated with higher levels of harmful alcohol use and later burnout complaints (Mento et al., 2020; O’Brien et al., 2024; Rossi et al., 2023). Notably, the positive association between violence and harmful alcohol use was most apparent under weak leadership conditions and diminished at average and stronger levels of leadership climate. Although the indirect effect was small, its emergence primarily under weaker leadership conditions underscores the role of leadership climate in shaping how employees interpret and respond to stressful or threatening encounters. The pattern suggests that harmful alcohol use may function as a behavioural strain response under insufficient leadership support, in line with coping and self-medication theories (Teo et al., 2020). At the same time, the small magnitude should be interpreted with caution and reflects the multifactorial nature of burnout, which may also be influenced by numerous organisational, interpersonal, and personal factors.

From a Job Demands–Resources (JD–R) perspective (Bakker et al. 2023; Dollard and Bakker 2010), these findings contribute to the framework by illustrating a potential behavioural pathway through which a job demand (workplace violence) may become associated with later strain outcomes. Specifically, harmful alcohol use may represent one coping-related mechanism linking exposure to workplace violence with subsequent burnout complaints, whereas leadership climate appears to function as an organisational resource that may modestly weaken this pathway. In this way, the findings extend JD–R theory by highlighting how organisational resources may shape not only direct stress–strain relationships but also behavioural responses to workplace demands.

These results also align with previous evidence highlighting the role of psychosocial safety climate and supportive leadership in buffering stress responses and promoting resilience in healthcare settings (McLinton et al., 2019; Spence Laschinger et al., 2009). They also emphasise the importance of understanding how specific leadership behaviours shape employees’ psychological reactions to violent or threatening encounters.

Exploratory profession-stratified analyses suggested that the moderated mediation pattern was observed among nurses but not physicians. Among nurses, leadership climate significantly moderated the association between violence and harmful alcohol use, and the index of moderated mediation was statistically significant, suggesting that the strength of the indirect association between violence and burnout via harmful alcohol use varied as a function of leadership climate. Among physicians, neither the interaction nor the indirect effect reached significance, although violence continued to show a direct association with burnout complaints. One possible explanation is that nurses experience more frequent patient contact, emotional labour, and exposure to aggression, and may depend more heavily on unit-level leadership for support and protection (Arnetz and Arnetz, 2001; Fernemark et al., 2024). Physicians, in contrast, often work with greater autonomy and may rely less on daily leadership for coping, which may explain the absence of moderated pathways. This divergence highlights the value of assessing leadership processes within distinct professional subgroups rather than assuming uniform effects across healthcare occupations. However, formal interaction tests did not support statistically significant occupational differences. Accordingly, these subgroup findings should be interpreted cautiously and viewed as hypothesis-generating rather than confirmatory.

Together, these findings advance the literature on workplace violence and protective organisational factors by demonstrating that leadership climate shapes behavioural coping responses to workplace violence, and that supportive leadership may modestly weaken a pathway from violence to later burnout via harmful drinking, with broadly similar patterns across professional groups. Rather than indicating strong protective effects, the findings suggest a modest but theoretically consistent buffering pattern. Leadership appears to play a role in mitigating behavioural and emotional strain following exposure to violence, particularly for nurses and frontline staff.

## 5 Practical implications

The results underscore the value of strengthening leadership climate within healthcare organisations, particularly in units with higher exposure to aggression. Leadership development interventions that promote supportive communication, participatory decision-making, fairness, and proactive follow-up after violent incidents may help reduce reliance on maladaptive coping behaviours and support employees' mental health. Given that these buffering effects were more apparent among nurses, tailored initiatives focused on unit-level leadership, supervisory availability, and psychological safety may be especially effective in nursing contexts (Ystaas et al., 2023). Organisational policies that encourage incident reporting, provide structured debriefing, and ensure access to support resources may further contribute to a safer and more supportive work environment (Yang et al., 2018).

Importantly, our findings suggest that even relatively modest statistical effects may be meaningful when they operate across large healthcare organisations and over time. Small indirect pathways via harmful alcohol use could signal “early warning signs” of strain in staff groups, offering an opportunity for preventive action before more severe burnout or turnover develops. This aligns with previous work in occupational health psychology, showing that small effect sizes are common but cumulative in their impact on health and performance (Brauchli et al., 2015).

## 6 Strengths, limitations, and future research

This study has several strengths. First, it draws on a large, nationally representative longitudinal cohort of healthcare workers, thereby enhancing the generalizability of the findings within the Swedish healthcare context. Second, the prospective design, with temporal separation between antecedents and outcomes, allows examination of associations over time. Third, the inclusion of both physicians and nurses allowed for profession-specific analyses, providing insight into how organisational resources such as leadership climate may operate differently across occupational groups.

Several limitations should be acknowledged. First, all data were based on self-reports, which may introduce common-method bias and social desirability, particularly for sensitive domains such as alcohol use and experiences of violence (Podsakoff et al., 2003). In addition, because workplace violence, leadership climate, and harmful alcohol use were all assessed at T1, the indirect pathway does not have full temporal separation and should be interpreted as a conditional indirect prospective association rather than a causal process (Maxwell and Cole, 2007). Recent longitudinal research has further emphasised the importance of stronger temporal designs and more differentiated assessments of workplace violence to better capture dynamic processes and potential causal pathways (Zhang et al., 2025).

Second, the response rate was modest, which is common in large-scale occupational surveys, but nonetheless raises the possibility of nonresponse bias and sample selection (Groves, 2006). Participants who remained in the analytic sample may differ systematically from nonparticipants or dropouts, for example, in terms of health status, workload, or coping resources. In addition, the relatively high proportion of experienced workers suggests potential survivorship effects, which may limit generalisability and attenuate observed associations.

Third, participants with high burnout complaints at T1 were excluded in order to focus on prospective associations among workers not already in the high-risk range. This approach reduced the likelihood that observed associations reflected continuation of existing burnout or reverse causation, thereby strengthening the interpretation of prospective risk pathways. However, this choice limits generalisability and precludes conclusions about violence-related processes among workers with established burnout.

Fourth, although the longitudinal design strengthens temporal interpretation, causal inference remains limited. Fifth, the explained variance in harmful alcohol use and burnout was modest, which is typical in psychosocial occupational research but nonetheless suggests that additional individual and organisational factors may contribute to these outcomes (Judge et al., 2002). More recent evidence suggests that exposure to workplace violence and related psychosocial conditions remain key predictors of burnout, underscoring the need to incorporate a broader range of contextual variables (O’Brien et al., 2024).

Sixth, workplace violence was dichotomised in the main analyses, which simplifies interpretation but may reduce information on frequency (MacCallum et al., 2002). Sensitivity analyses using the original four‑category measure yielded a broadly similar pattern of results, although effect estimates—particularly conditional indirect effects—were attenuated. This suggests some sensitivity of the findings to the operationalisation of workplace violence. Given that the four response categories are ordinal, lack meaningful interval properties, and are highly skewed, results from the sensitivity analyses should be interpreted as robustness checks rather than definitive dose–response estimates.

Finally, although we adjusted for key covariates, residual confounding cannot be ruled out. Future research should consider factors such as baseline alcohol use, social support, psychological distress, and trauma history, which may influence both exposure to workplace violence and subsequent outcomes. Future research may also benefit from examining alternative coping responses beyond alcohol use (Halbesleben and Buckley, 2004), different leadership approaches (e.g., empowering or trauma-informed leadership) (Eva et al. 2019), and multilevel designs that capture how unit-level context shapes responses to workplace violence (González-Romá and Hernández, 2017).

## 7 Conclusions

Taken together, this study suggests that workplace violence is associated with harmful alcohol use and later burnout. It further shows that leadership climate may modestly shape the prospective associations between workplace violence, maladaptive coping through harmful alcohol use, and subsequent burnout complaints among healthcare workers in Sweden. The findings were broadly consistent with the view that supportive leadership constitutes an organisational resource in high-strain work settings, particularly in contexts where staff are exposed to threats or violence. However, the effects were small, the indirect pathway lacked full temporal separation, and the results should therefore be interpreted cautiously. Strengthening leadership practices may represent a potentially useful avenue for supporting healthier coping responses, reducing strain, and mitigating longer-term burnout risk.

## Declaration of AI-assisted technologies in the manuscript preparation process

During the preparation of this manuscript, the authors used a paid Grammarly subscription to review English grammar, spelling, and language clarity. The tool was used solely for language editing purposes and did not contribute to the study design, data analysis, interpretation of results, or substantive content. The authors reviewed and edited all text following its use and take full responsibility for the content of the published article.

## Funding

This study was funded by Afa Insurance (#220,177).

## CRediT authorship contribution statement

**Josefina Peláez Zuberbuhler:** Writing – review & editing, Writing – original draft, Methodology, Formal analysis, Data curation, Conceptualization. **Emelie Thern:** Writing – review & editing, Conceptualization. **Bodil J. Landstad:** Writing – review & editing, Conceptualization. **Malin Sjöström:** Writing – review & editing, Conceptualization. **Emma Brulin:** Writing – review & editing, Writing – original draft, Supervision, Software, Resources, Project administration, Methodology, Investigation, Funding acquisition, Formal analysis, Data curation, Conceptualization.

## Declaration of competing interest

The authors declare that they have no known competing financial interests or personal relationships that could have appeared to influence the work reported in this paper.
